# Psychological mechanisms underlying employability among Chinese university students: a sequential mediation model and gender invariance analysis

**DOI:** 10.3389/fpsyg.2026.1749175

**Published:** 2026-03-11

**Authors:** Hongying Chang, Guohua Yan, Jichang Guo

**Affiliations:** 1 Department of Psychology, School of Educational Sciences, Minzu Normal University of Xingyi, Xingyi, China; 2 Department of History, School of Humanities, Anshun University, Anshun, China

**Keywords:** capital conversion model, future employability, higher education intervention, measurement invariance, sequential mediation

## Abstract

**Background:**

Addressing intensified labor market competition, this study validates the multidimensional structure of future employability and examines its underlying sequential capital conversion process-a critical objective for higher education-within a non-Western cultural context.

**Methods:**

Data from 1,304 Chinese undergraduates were analyzed using a rigorous structural modeling approach: Confirmatory Factor Analysis (CFA), Multi-Group CFA (MG-CFA) to establish Measurement Invariance (MI) across gender, and Sequential Structural Equation Modeling (Sequential SEM). The validated four-factor model comprises Human Capital, Psychological Capital, Social Capital, and Career Development. The successful MI test permitted robust latent mean comparisons.

**Results:**

Multi-group analyses revealed significant gender differences in resource possession: males excelled in Human Capital, while females reported significantly higher latent means in Psychological Capital. The sequential mediation analysis demonstrated that Human Capital significantly influenced Career Development through the sequential mediating roles of Psychological Capital and Social Capital. Crucially, Psychological Capital was identified as the pivotal psychological engine in this conversion process, serving as the foundational resource that facilitates the mobilization of social capital and subsequent career development.

**Conclusion:**

This research provides robust empirical support for a dynamic, integrated capital conversion model. Theoretically, it advances capital theory by demonstrating the sequential interplay between cognitive, personality, and social factors, underscoring Psychological Capital as the pivotal engine that facilitates this conversion process. Practically, the findings suggest that higher education interventions should prioritize psychological resilience and self-efficacy alongside traditional skill-building to design gender-sensitive programs that optimize resource transformation for sustainable employability.

## 1 Introduction

In the contemporary, rapidly evolving technological landscape—characterized by profound digital transformation and global economic structural shifts—university students' employability has surfaced as a paramount indicator of higher education institutional effectiveness on a global scale. A substantial body of evidence underscores that, within increasingly volatile and non-linear labor markets, employability serves as a paradigmatic benchmark for evaluating the institutional success of higher education institutions (HEIs) (Eimer and Bohndick, 2023; Scandurra et al., 2024). Against this backdrop, future employability—conceptualized as an individual's capacity to secure, sustain, and attain fulfilling employment within fluid labor markets (Fugate et al., 2004)—has progressed beyond a mere personal developmental objective to become a foundational metric of HEIs' societal function. However, as labor market dynamics transition from credential-based to competency-driven paradigms, conventional linear career trajectories are increasingly destabilized, subjecting graduates to intensified professional uncertainty and transitional strain (Donald et al., 2023; Petruzziello et al., 2024).

This predicament is particularly pronounced and multifaceted within the Chinese socio-economic context. Over the preceding decade, China's higher education sector has undergone unprecedented expansion, resulting in an annual influx of millions of graduates into the labor market. This demographic surge has exacerbated labor market competition, catalyzing a phenomenon academically identified as “intensified positional competition,” or colloquially termed “neijuan” (involution). Structural discrepancies between the extensive supply of graduates and the sophisticated demand for multidimensional talent have progressively eroded the “credential premium,” rendering the competition for high-tier positions exceptionally rigorous (Xiang et al., 2023; Yao et al., 2023; Scandurra et al., 2024). In such an adversarial environment, reliance on monolithic knowledge reserves is increasingly inadequate; emerging empirical evidence suggests that individuals must proactively mobilize both psychological and social resources to achieve professional breakthroughs amidst these structural constraints (Chen et al., 2023; Letnar and Širok, 2025).

The scholarly conceptualization of employability is undergoing a significant paradigm shift, transcending narrow skill-centric frameworks toward a dynamic, multidimensional system that integrates human capital (knowledge and skills), psychological capital (personal qualities), and social capital (social networks) (Eimer and Bohndick, 2023; Petruzziello et al., 2024; Xu et al., 2022). This integrative perspective posits that employability emanates from the synergistic interplay between technical competencies, intrinsic motivational states, and extrinsic network support (Letnar and Širok, 2025; O'Donovan et al., 2024). Nevertheless, existing literature predominantly provides static, cross-sectional observations of these dimensions, emphasizing discrete contributions rather than inter-dimensional, synergistic transformations (Fang and Xu, 2025). Specifically, the mechanisms through which knowledge and skills are internalized into psychological resilience and subsequently externalized into social assets remain insufficiently theorized. Contemporary theoretical advancements have begun postulating the necessity of exploring the dynamic conversion mechanisms of employability capital; however, empirical substantiation remains sparse, particularly within non-Western sociocultural contexts (Petruzziello et al., 2024; Donald et al., 2023). Neglecting these micro-level conversion processes has resulted in theoretical lacunae, often predisposing higher education interventions toward an overemphasis on vocational training while marginalizing psychological empowerment.

### 1.1 Theoretical foundation: the sequential logic of capital conversion

Although the multidimensional architecture of employability has been preliminarily delineated, prior empirical investigations largely presuppose that its constituent dimensions are conceptually atomized and static (Petruzziello et al., 2024; Eimer and Bohndick, 2023). Such a paradigm neglects the processual nature of how employability capital interacts and accrues value throughout the professionalization trajectory. The present study introduces the Sequential Capital Conversion Model, which seeks to elevate the conceptualization of employability from a static “checklist of traits” to a dynamic resource mobilization mechanism.

This theoretical logic is anchored in Social Cognitive Career Theory (SCCT) (Lent et al., 1994; Lent and Brown, 2019), which asserts the centrality of individual cognition in resource cultivation. Environmental resources do not unilaterally dictate outcomes; rather, their effects are mediated via internal psychological processes. Accordingly, while human capital (X) constitutes the foundational resource, its efficacy is fundamentally contingent upon the individual's capacity to internalize objective competencies into psychological resources and subsequently externalize them into social capital (Bandura, 1997; Luthans et al., 2015; Youssef-Morgan, 2024). This flow exemplifies the conversion of capital from potential to kinetic energy: accumulated knowledge reconfigures psychological dispositions, and augmented psychological resources serve as a relational engine, empowering individuals to navigate intricate social ecologies and leverage critical information and opportunities (Dóci et al., 2023; Fang and Xu, 2025).

### 1.2 Hypotheses development: a cascading mechanism

Synthesizing the tenets of SCCT (Lent et al., 1994; Lent and Brown, 2019), this study delineates a sequential mediation pathway from human capital to career outcomes, adhering to a cascading logic: “internalization reifies traits → traits catalyze behavior → behavior manifests value.” SCCT maintains that career outcomes are not direct derivatives of environmental factors or innate ability but are incrementally generated through a sequence of psychological and behavioral iterations, offering a robust theoretical scaffold for examining the dynamic conversion among employability capitals.

First, the internalization of human capital into psychological capital (X → M1). The mastery of knowledge and skills serves as the prerequisite for shaping psychological dispositions. Consistent with self-efficacy theory (Bandura, 1997), mastery experiences and skill acquisition significantly augment individuals' perceived agency over future career exigencies, progressively internalizing these objective gains into stable psychological assets such as confidence, resilience, and proactivity (Luthans et al., 2015; Youssef-Morgan, 2024). In hyper-competitive and volatile employment settings, robust professional competence not only provides instrumental utility but also underpins psychological equilibrium and positive future-oriented expectancies. Contemporary empirical evidence further identifies human capital as a vital antecedent of psychological capital formation, particularly during the formative stages of early-career development (Ma et al., 2024; Petruzziello et al., 2024). Accordingly, we propose:

**Hypothesis 1:** Human capital (knowledge and skills) positively predicts psychological capital (personal qualities).

Second, the mobilizing effect of psychological capital on social capital (M1 → M2). Individuals' intrinsic psychological dispositions profoundly influence their interactions with external social environments. Those endowed with higher psychological capital typically demonstrate greater initiative and adaptive, forward-looking behaviors, thereby facilitating the cultivation and maintenance of high-tier social networks (Van der Heijden et al., 2009; Dóci et al., 2023). Psychological capital mitigates social anxiety and uncertainty during interpersonal interactions while enhancing the capacity to navigate and sustain relationships within complex relational structures. Recent inquiries highlight its function as an activator of social capital, providing the requisite psychological substrate for network expansion and operational functionality (O'Donovan et al., 2024). Accordingly, we propose:

**Hypothesis 2:** Psychological capital (personal qualities) positively predicts social capital (social networks).

Finally, the cascading conversion from X → M1 → M2 → Y. Social capital, by facilitating access to critical information, emotional support, and opportunity conduits, ultimately catalyzes professional development (Letnar and Širok, 2025; O'Donovan et al., 2024). Conventional parallel models, which treat human, psychological, and social capital as independent predictors, fail to elucidate why career outcomes diverge significantly among individuals with equivalent skill sets (Fang and Xu, 2025). By integrating a sequential logic, this study maintains that human capital must first enhance career readiness via psychological internalization and subsequently realize resource externalization through social mobilization. In other words, career success is not the direct product of a singular capital dimension but the cumulative result of multi-capital sequential conversion. Accordingly, we propose:

**Hypothesis 3:** Psychological and social capital sequentially mediate the relationship between human capital (knowledge and skills) and career development.

### 1.3 The present study: integrating contextual nuances and methodological rigor

Within the context of China's rapidly diversifying economy and fiercely competitive labor market, empirical testing of the Sequential Capital Conversion Model holds substantial theoretical and utilitarian significance. Given the persistent expansion of higher education and the concomitant erosion of credential premiums, university students face challenges that have shifted from mere “qualification acquisition” to the effective integration and mobilization of heterogeneous resources (Chen et al., 2023; Scandurra et al., 2024). In these circumstances, purely skill-centric paradigms are insufficient, highlighting the imperative to investigate the nuanced roles of psychological and social resources in professional trajectories.

To capture the micro-level logic of employability capital—from internalization to externalization—this study analyzes data from 1,304 undergraduates across multiple Chinese metropolitan areas, utilizing a rigorous methodological framework. First, confirmatory factor analysis (CFA) was executed to validate the construct integrity of multidimensional employability. Second, measurement invariance (MI) and multi-group CFA (MG-CFA) were utilized to ensure cross-group metric equivalence between genders and to mitigate potential measurement bias, adhering to established psychometric conventions (Putnick and Bornstein, 2016; Leitgöb et al., 2023; Lasker, 2024). Finally, sequential structural equation modeling (SEM) integrated with bias-corrected bootstrapping was applied to empirically test the cascading mediation effects, thereby enhancing the robustness of the causal path estimations (Alfons et al., 2022).

**Figure 1:**
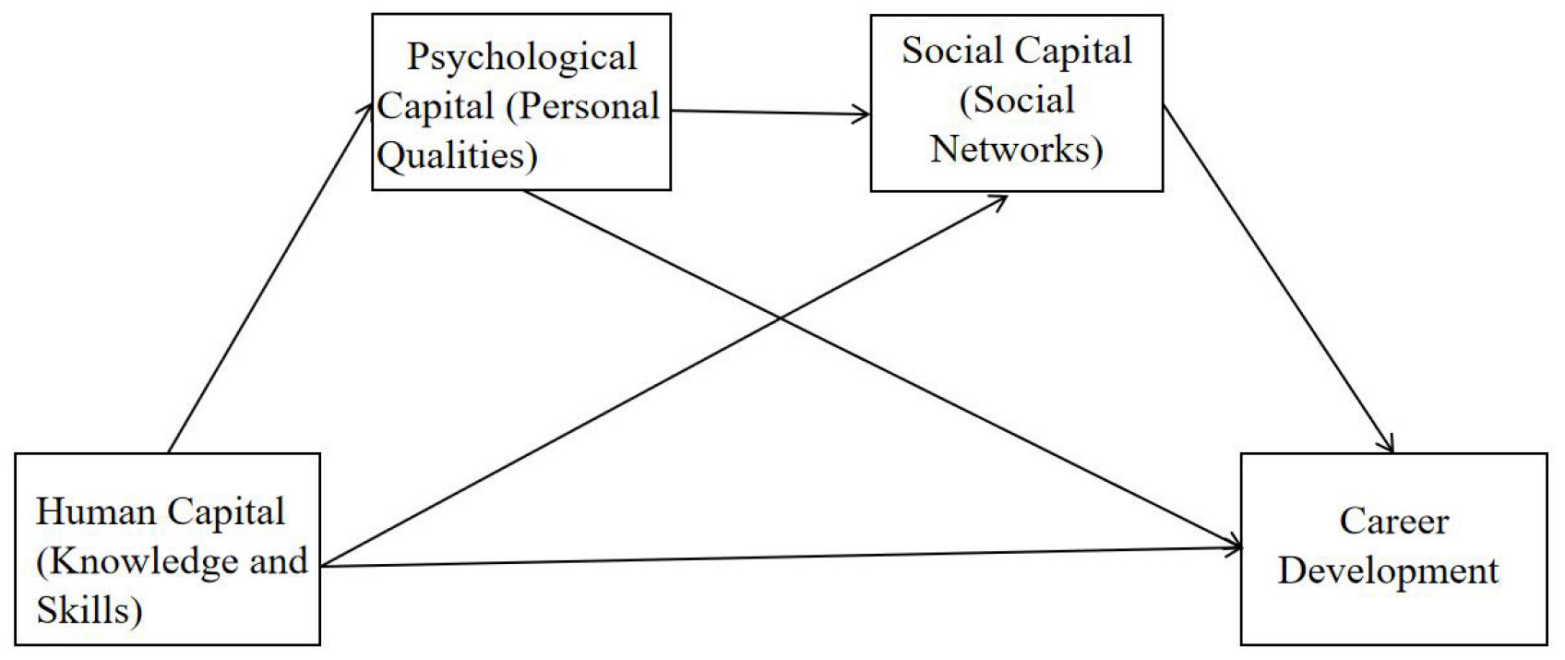
The theoretical model of multiple mediators. For clarity, the measurement model (indicator variables and error terms) is omitted.

Through this integrative design, the study contributes to employability research on several fronts. Theoretically, it empirically substantiates the X → M1 → M2 → Y sequential conversion pathway, synthesizing psychological, educational, and sociological perspectives while addressing the enduring question of how psychological assets are transformed into career-related resources. The conceptual framework of this study is illustrated in Figure 1. Methodologically, the establishment of scalar invariance provides a robust statistical platform for gender-based comparisons within the Chinese context and offers a template for increasing precision and cross-group comparability in future employability research. Most critically, the findings provide evidence-based insights for HEIs to implement a “skills–psychological–social” triadic framework, empowering graduates to achieve resilient career development in highly volatile labor markets.

## 2 Methods

### 2.1 Participants

A stratified convenience sampling approach was employed to recruit participants from universities situated across four distinct inland cities in China (Yibin, Shaoguan, Anshun, and Xingyi). Data collection was administered online using the professional survey platform “Wenjuanxing” (Questionnaire Star). This specific regional selection was strategically implemented to capture the experiences of graduates confronting structural disparities and heightened resource competition, which are characteristic of non-tier-one urban labor markets (Eimer and Bohndick, 2023). This contextual choice directly addresses the issue of involution (neijuan) highlighted in the Introduction. A total of 1,450 questionnaires were initially returned. Following standard data screening protocols, responses were filtered for quality, resulting in the exclusion of 146 cases due to insufficient completion time or failure to pass embedded attention checks. The final valid sample consisted of 1,304 participants, yielding an effective response rate of 89.93%.

The demographic characteristics of the valid sample were as follows: The sample exhibited a well-balanced gender distribution, comprising 646 male (49.54%) and 658 female (50.46%) students. By academic year, the distribution was 390 first-year students (29.91%), 353 second-year (27.08%), 272 third-year (20.86%), and 289 fourth-year (22.16%).

This sample size exceeds widely accepted recommendations for structural equation modeling in survey research and ensures strong statistical power for model evaluation. Methodological authorities suggest that adequate SEM sample size typically ranges from 200 to 500 depending on model complexity (Kline, 2016). More rigorous Monte Carlo simulation research indicates that sample sizes above 1,000 substantially reduce estimation bias and protect solution propriety in complex mediation models (Wolf et al., 2013). Similarly, power analysis work has demonstrated that larger samples significantly increase the likelihood of detecting meaningful effects in covariance structure modeling (MacCallum et al., 1996). Practical guidelines for survey-based SEM further support that samples exceeding 500 are preferable for robust parameter estimation (Memon et al., 2020). Thus, the current sample of 1,304 participants provides more than adequate statistical power for the multi-group SEM and sequential mediation analyses conducted in this study.

### 2.2 Measures

The Future Employability Scale for Chinese College Students (Chen et al., 2023) was administered to assess participants' self-perceived employability. The instrument is comprised of 28 items, which delineate the four core dimensions of capital:

(1) Knowledge and Skills (Human Capital), (2) Personal Qualities (Psychological Capital), (3) Social Networks (Social Capital), and (4) Career Development (Outcome).

All items are framed as positive statements and rated on a five-point Likert scale (1 = strongly disagree, 5 = strongly agree), where a higher aggregate score signifies a stronger perception of future employability.

In the present study, the overall internal consistency reliability was excellent (Cronbach's α = 0.923). Subscale reliabilities ranged from 0.715 to 0.832, comfortably meeting the commonly accepted criteria for psychological measurement tools (Nunnally and Bernstein, 1994). These findings collectively indicate that the scale possesses satisfactory psychometric properties within this specific sample.

### 2.3 Structural model specification and path analysis

The validated 27-item measurement model was utilized to specify the hypothesized sequential mediation structural equation model (SEM). The model was empirically grounded in the theoretical premise of capital conversion, whereby Knowledge and Skills (X) was defined as the exogenous predictor of Career Development (Y), with Personal Qualities (M1) and Social Networks (M2) functioning as sequential mediators (X → M1 → M2 → Y). The model was estimated using Maximum Likelihood (ML) estimation with robust standard errors (if applicable) in Mplus 8.0 (Muthén and Muthén, 2017).

To formally test the statistical significance of the indirect effects, the bias-corrected percentile bootstrap method with 5,000 resamples was applied (Hayes, 2018). This non-parametric approach is widely preferred because it bypasses the restrictive assumption of normality in the sampling distribution of the indirect effect, thereby enhancing the robustness of the confidence intervals (Alfons et al., 2022; Hayes, 2018). An indirect effect was considered statistically significant when the 95% confidence interval (CI) did not include zero.

### 2.4 Common method bias test

Given that all data were collected via self-report measures at a single time point, the potential for Common Method Bias (CMB) was a concern. Both procedural and statistical controls were implemented to proactively address this threat (Podsakoff et al., 2003). Procedurally, the anonymity of all responses was guaranteed, and measures of the predictor, mediators, and outcome variables were spatially separated into distinct sections to mitigate cognitive contamination. Statistically, Harman's single factor test was applied as an initial, preliminary check. The unrotated principal components analysis indicated that the first factor accounted for 18.41% of the total variance, well below the conservative 40% threshold typically cited as a serious threat. Furthermore, a Confirmatory Factor Analysis (CFA) incorporating an unmeasured latent method factor (ULMF) was conducted (Williams et al., 2010). The fit of the ULMF model (χ^2^/df =3.15, CFI = 0.91, TLI = 0.90, RMSEA = 0.048) demonstrated only a marginal improvement over the original four-factor model, and the factor loadings remained notably stable. These converging results strongly suggest that CMB does not constitute a serious threat to the validity and interpretation of the findings.

## 3 Results

### 3.1 Descriptive statistics, reliability, and validity

Table 1 presents the descriptive statistics (means and standard deviations), internal consistency estimates (Cronbach's α), and the intercorrelation matrix for all latent constructs (*N* = 1,304).

**Table 1:** Descriptive statistics, reliability, and intercorrelations.

**Variable**	***M***	***SD***	**α**	**1**	**2**	**3**	**4**
1.Knowledge and skill	3.49	0.54	0.832				
2.Personal qualities	3.74	0.52	0.825	0.619^***^	–		
3.Social networks	3.58	0.54	0.828	0.597^***^	0.663^***^	–	
4.Career development	3.49	0.57	0.715	0.624^***^	0.560^***^	0.683^***^	–

^***^p < 0.001. Means and SDs reported to two decimal places. Subscale scores reflect the average of items composing each dimension.

*Reliability*. The internal consistency for each latent construct was deemed acceptable to excellent: Cronbach's α values ranged from 0.715 to 0.832, thereby exceeding the conventionally accepted threshold of 0.70 (Nunnally and Bernstein, 1994). The overall scale demonstrated excellent reliability (α = 0.923). These results confirm the satisfactory internal consistency required for the subsequent multivariate analyses.

*Descriptive statistics*. Subscale means (on a 1–5 Likert scale) ranged from *M* = 3.49 to 3.74, with standard deviations ranging from SD = 0.50 and SD = 0.60. These values indicate moderate-to-high average endorsement levels and acceptable data dispersion within the sample.

*Intercorrelations*. All pairwise correlations among the four dimensions were positive and statistically significant at *p* < 0.001. Correlation coefficients ranged from *r* = 0.597 (Personal Qualities with Social Networks) to *r* = 0.683 (Personal Qualities with Career Development). The presence of these significant associations among predictors, mediators, and outcome is consistent with the necessary prerequisites for sequential mediation analysis (Hayes, 2013) and provides a solid statistical foundation for testing the proposed mediation model.

### 3.2 Confirmatory factor analysis (CFA)

To rigorously evaluate the structural validity of the Chinese College Students' Future Employability Scale, Confirmatory Factor Analysis (CFA) was performed to test the fit of the hypothesized four-factor model.

*Initial CFA*. The initial four-factor CFA (comprising 28 items) yielded the following fit indices: χ^2^_(344)_ = 1,450.995, *p* < 0.001; RMSEA = 0.050 [90% CI (0.047, 0.052)]; CFI = 0.918; TLI = 0.910; SRMR = 0.040. While the χ^2^ test was significant (a common finding in large samples; Bentler, 1990), the alternative fit indices met commonly accepted criteria for acceptable to good fit (RMSEA < 0.08, CFI/TLI > 0.90, SRMR < 0.08) (Hu and Bentler, 1999), strongly supporting the proposed four-factor specification.

*Factor loadings and item diagnostics*. Standardized factor loadings for the majority of items ranged between 0.55 and 0.73, thus exceeding the widely recommended cutoff of 0.50 for practical significance (Hair et al., 2010) and indicating that most items adequately reflect their intended latent constructs. Notably, one item (Item 26, belonging to the Career Development factor) exhibited a standardized loading below 0.50 and concurrently displayed an elevated modification index, suggesting low measurement purity and potential model misspecification introduced by that specific item.

#### 3.2.1 Scale refinement and model parsimony

Guided by the principle of maximizing parsimony while maintaining measurement validity (Anderson and Gerbing, 1988), the impact of removing Item D26 on both model fit and construct integrity was assessed. Following the deletion of D26, the revised 27-item four-factor model demonstrated markedly improved fit: χ^2^/df = 4.09, RMSEA = 0.049, CFI = 0.923, TLI = 0.915, SRMR = 0.038. The decrease in RMSEA and the corresponding increase in CFI indicate a meaningful statistical improvement in model fit, while the χ^2^/df remained < 5, consistent with acceptable model parsimony (Marsh and Hocevar, 1985).

In light of these psychometric diagnostics and the theoretical evaluation of item content, the 28-item scale was retained for all subsequent analyses. The removal of Item 27 enhanced the psychometric coherence of the Career Development factor without materially altering the substantive interpretation of the construct.

### 3.3 Measurement invariance (MI) across gender

Next, the equivalence of the four-factor structure of the Future Employability Scale across male and female participants was assessed using Multi-Group Confirmatory Factor Analysis (MG-CFA). The invariance testing proceeded sequentially from configural (testing the same factor-item pattern) to metric (testing equal factor loadings) and finally to scalar (testing equal item intercepts) invariance. The fit statistics for each sequential step are summarized in Table 2.

**Table 2:** MG-CFA.

**Model**	***χ^2^***	**df**	***RMSEA***	***CFI***	***TLI***	***SRMR***	***ΔCFI***	***ΔTLI***	***ΔRMSEA***
Configural	1,858.29	636	0.054	0.910	0.901	0.044			
Metric	1,889.42	659	0.054	0.910	0.904	0.049	0.000	0.003	−0.005
Scalar	1,943.98	682	0.053	0.907	0.905	0.050	−0.003	0.001	0.001

Although the χ^2^ difference tests indicated non-trivial χ^2^ changes when constraints were imposed (Δχ^2^ = 31.13, Δdf = 23, *p* > 0.05 for metric; Δχ^2^ = 54.56, Δdf = 23, *p* < 0.01 for scalar), We relied on changes in practical fit indices (ΔCFI ≤ 0.01; ΔRMSEA ≤ 0.015) as recommended in the measurement invariance literature (Cheung and Rensvold, 2002; Chen, 2007). The observed ΔCFI and ΔRMSEA values all met commonly used thresholds (ΔCFI ≤ 0.01; ΔRMSEA ≤ 0.015), indicating that imposing equality constraints on loadings and intercepts did not meaningfully degrade model fit. Therefore, the scale demonstrated configural, metric, and scalar invariance across gender.

*Interpretation*. These results indicate that (a) the same four-factor configuration is represented in both sexes, (b) items relate to latent constructs to a comparable degree across sexes, and (c) item intercepts are comparable-together permitting meaningful comparison of latent means between male and female groups (Cheung and Rensvold, 2002; Chen, 2007).

#### 3.3.1 Latent mean differences (gender)

Given the establishment of scalar invariance, latent means were then formally compared between females and males (with the male mean fixed to zero for reference). Results indicated small but statistically significant gender differences on two latent constructs:

Knowledge and skills: the female mean was −0.124 [SE = 0.061, *p* = 0.043, 95% CI (−0.244, −0.004)], indicating that females reported slightly lower latent scores than males.

Personal qualities: the female mean was 0.131 [SE = 0.064, *p* = 0.041, 95% CI (0.005, 0.257)], indicating that females reported slightly higher latent scores than males.

Social networks: the female mean was −0.042 [SE = 0.062, *p* = 0.499, 95% CI (−0.163, 0.079)], demonstrating no statistically significant difference.

Career development: the female mean was −0.094 [SE = 0.064, *p* = 0.145, 95% CI (−0.219, 0.031)], demonstrating no statistically significant difference.

*Interpretation*. The refined scale supports valid cross-gender comparisons: males reported marginally higher perceived knowledge and skills, whereas females reported marginally higher personal qualities. No gender differences were observed for social networks or anticipated career development. These nuanced differences, while small in magnitude, carry the potential to inform gender-sensitive approaches in career education and counseling.

### 3.4 Structural model and path analysis

A sequential mediation model was tested, in which Knowledge and Skills (X) predicted Career Development (Y), with Personal Qualities (M1) and Social Networks (M2) specified as first- and second-stage mediators (X → M1 → M2 → Y). Indirect effects were estimated using a bias-corrected percentile bootstrap method with 5,000 resamples (Hayes, 2013).

To examine the statistical significance of the indirect effects, the bias-corrected percentile bootstrap method with 5,000 resamples was applied (Hayes, 2013; Preacher and Hayes, 2008). This non-parametric procedure is considered superior to traditional causal-step approaches (e.g., Baron and Kenny, 1986), as it does not rely on the restrictive assumption of normality in the sampling distribution of indirect effects and provides more robust confidence intervals (CIs) under conditions of non-normality (Alfons et al., 2022). The indirect effect was regarded as statistically significant when the 95% confidence interval did not include zero.

#### 3.4.1 Measurement model validity

The measurement model utilized within the structural analyses exhibited acceptable fit: $\chi_{\left(340\right)}^{2}$ = 1,548.11, *p* < 0.001; CFI = 0.919; TLI = 0.909; RMSEA = 0.052 [90% CI (0.049, 0.054)]; SRMR = 0.040. Standardized factor loadings were all statistically significant (*p* < 0.001) and ranged from 0.552 to 0.724. Composite Reliabilities (CRs) ranged from 0.87 to 0.92, and Average Variance Extracted (AVE) values ranged from 0.50 to 0.58, collectively supporting convergent validity; discriminant validity was also successfully supported by the Fornell–Larcker criteria (Fornell and Larcker, 1981; Hair et al., 2010) (refer to Table 3 for CR, AVE, and item loadings.)

**Table 3:** CR, AVE, and item loadings.

**Latent variable**	**Scope of question types**	**Item loadings**	**CR**	**AVE**
Knowledge and skill	A1–A7	0.602–0.724	0.92	0.58
Personal qualities	A8–A14	0.573–0.690	0.89	0.53
Social networks	A15–A21	0.553–0.691	0.88	0.50
Career development	A22–A27	0.596–0.668	0.87	0.52

#### 3.4.2 Structural paths and mediation results

**Figure 2:**
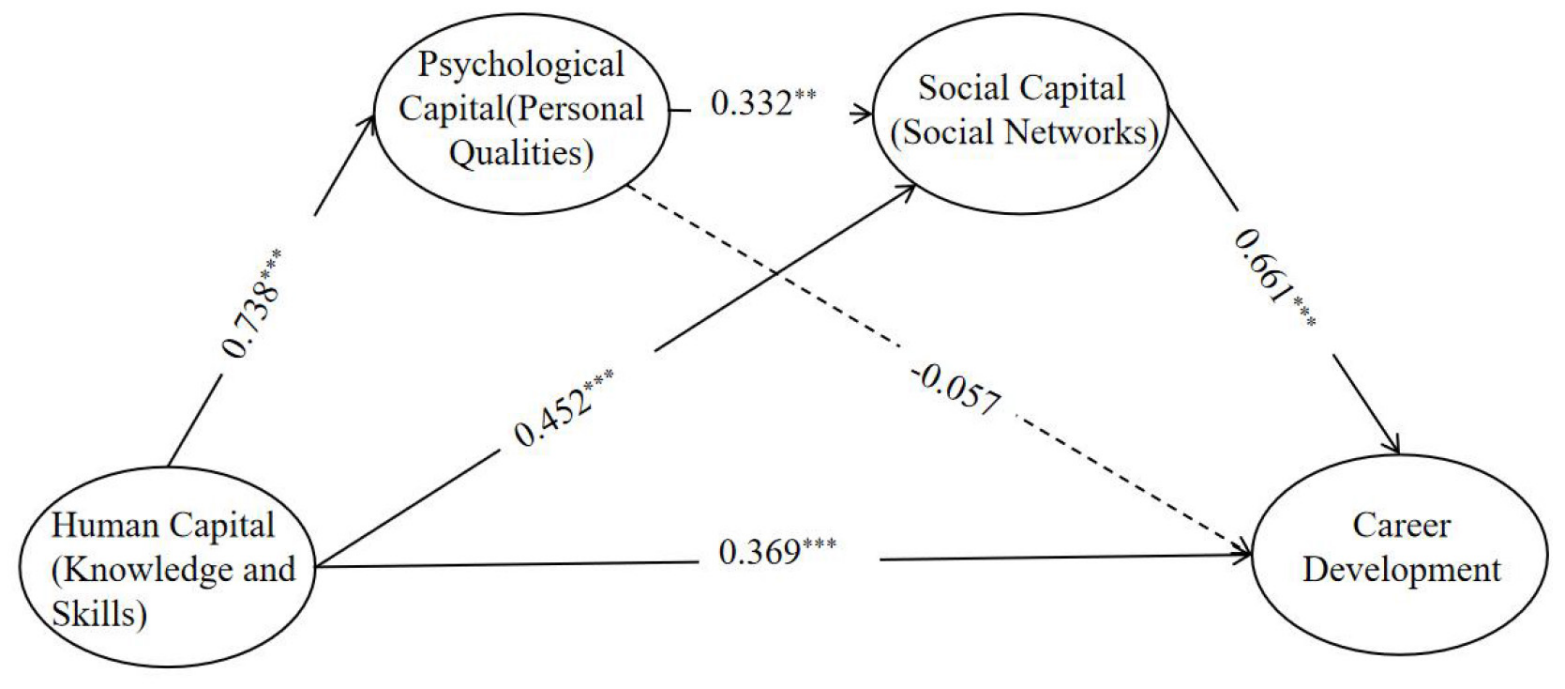
Sequential mediation model results. *N* = 1,304. Numbers on the paths represent standardized coefficients (β). Solid lines indicate significant paths at *p* < 0.001; the dashed line indicates a non-significant path. For clarity, the measurement model (indicator variables and error terms) is omitted. The corresponding detailed statistical estimates are provided in Table 4. **p < 0.01, ***p < 0.001

The final structural model demonstrated acceptable fit to the data, consistent with the fit reported in Table 3. The standardized path coefficients are detailed in Table 4. Knowledge and Skills (X) exhibited a significant positive direct effect on Career Development (Y) (β = 0.369, *p* < 0.001). Consistent with our sequential hypothesis, X significantly predicted Personal Qualities (M1), and M1 significantly predicted Social Networks (M2). Crucially, the direct path from Personal Qualities (M1) to Career Development (Y) was not statistically significant (β = −0.057, *p* = 0.349), providing evidence that the effect of M1 is fully routed through M2 (see Figure 2).

**Table 4:** Standardized path coefficients (with SE and 95% CI) from the final model.

**Variable relationships and effect types**	**Effect size**	**SE**	***t***	***p***	**95% CI**
**Path coefficient**					
X → M1	0.738	0.023	32.222	< 0.001	[0.692, 0.781]
M1 → M2	0.332	0.009	39.081	< 0.001	[0.314, 0.347]
X → M2	0.452	0.021	21.177	< 0.001	[0.407, 0.491]
M2 → Y	0.661	0.065	10.090	< 0.001	[0.539, 0.795]
M1 → Y	−0.057	0.061	−0.937	3.349	[−0.185, 0.056]
X → Y (direct)	0.369	0.054	6.891	< 0.001	[0.262, 0.472]
**Effect decomposition**					
X → Y (total)	0.831	0.046	14.548	< 0.001	[0.578,0.757]
X → Y (total indirect)	0.461	0.036	8.328	< 0.001	[0.395, 0.527]
Indirect 1: X → M2 → Y	0.299	0.036	8.328	< 0.001	[0.233, 0.374]
Indirect 2: X → M1 → M2 → Y	0.162	0.039	4.154	< 0.001	[0.088, 0.241]

Note on effect decomposition. The direct path from Personal Qualities to Career Development (M1 → Y) was not significant when Social Networks (M2) was included in the model, suggesting that Personal Qualities affect Career Development primarily via enhanced Social Networks. In other words, Personal Qualities appear to contribute to Career Development indirectly through their positive effect on Social Networks.

The total indirect effect of X on Y was significant (Effect = 0.461, SE = 0.036, *p* < 0.001), with its 95% bias-corrected confidence interval, 95% CI (0.395, 0.527), excluding zero. Both specific indirect effects were also statistically significant: (1) the indirect path through Social Networks (X → M2 → Y) was significant [Effect = 0.299, 95% CI (0.233, 0.374)]; and (2) the sequential indirect path through Personal Qualities and Social Networks (X → M1 → M2 → Y) was significant [Effect = 0.162, 95% CI (0.088, 0.241)]. The total effect of X on Y was 0.830 (0.369 direct + 0.461 indirect).

Knowledge and Skills exerts both direct and substantial indirect influences on anticipated Career Development. The indirect influence is notable, as a significant portion of X's effect on Y is transmitted through Social Networks (0.299) and, critically, via the sequential chain of Personal Qualities → Social Networks (0.162). These findings underscore the dual imperative of (a) equipping students with robust domain knowledge and skills and (b) fostering the necessary interpersonal resources and adaptive psychological traits that effectively promote networking-both of which are instrumental for students' perceived future career prospects.

## 4 Discussion

The present study empirically validates a cascading conversion mechanism through which Chinese university students' employability is transformed from human capital (X) into career development outcomes (Y) via the sequential mediation of psychological qualities (M1) and social networks (M2). By establishing this sequential conversion model, the study not only addresses the central inquiry regarding how capital is translated into career value—as raised in the Introduction—from a dynamic perspective, but also elucidates the underlying logic of individual resource circulation within a hyper-competitive (involutionary) labor market. This finding resonates with the contemporary paradigm shift in employability research, moving from a focus on static capital stocks toward dynamic capital flows and mobilization processes (Eimer and Bohndick, 2023; Letnar and Širok, 2025).

### 4.1 The sequential capital conversion mechanism

By constructing and empirically validating the X → M1 → M2 → Y pathway, this study uncovers the micro-level logic underlying employability capital circulation among Chinese university students. Confirmation of this cascading mechanism not only provides empirical substantiation for all proposed hypotheses but also offers a theoretical response to the core question of how capital transitions from a latent state to professional actualization, aligning with the foundational assumption of mobilizability in multi-capital employability frameworks (Fugate et al., 2004; O'Donovan et al., 2024).

First, the significant predictive effect of human capital on psychological qualities supports Hypothesis 1 and underscores the psychological empowerment function of skill accumulation in the professionalization process. In the context of China's diminishing credential premium, the monolithic possession of knowledge no longer generates substantial career returns through direct pathways (Xiang et al., 2023). Instead, the present findings suggest that the true value of human capital lies in its role as foundational fuel for psychological capital: a robust professional skill base can be effectively internalized into augmented career self-efficacy and resilience. This process of internalization corroborates psychological capital theory, which posits mastery experiences as a primary source of efficacy beliefs (Luthans et al., 2015; Youssef-Morgan, 2024), and aligns with recent empirical evidence from East Asian contexts indicating that human capital influences employment outcomes predominantly through psychological pathways (Pham et al., 2024).

Second, by validating the M1 → M2 → Y sequence (supporting Hypotheses 2 and 3), the study further clarifies how psychological resources are operationalized through social embeddedness. A particularly critical finding is the non-significant direct path from psychological qualities (M1) to career development (Y) (β = −0.057, *p* > 0.05). This statistical discontinuity indicates that internal psychological traits must undergo a crucial process of externalization to generate tangible career returns. According to the Theory of Planned Behavior (TPB), resources such as self-efficacy represent behavioral intentions or readiness rather than directly exchangeable labor-market “hard currency” (Ajzen, 1991). In China's guanxi-oriented job-search environment, psychological resources only become consequential when translated into proactive social behaviors—such as cultivating weak ties or engaging in informational interviews—through which individuals embed themselves within social networks that facilitate access to information and opportunities (Granovetter, 1973; De Schepper et al., 2023).

This finding strongly aligns with the assertion that social capital functions as a proximal converter within employability capital systems, serving as the key mechanism that translates internal potential into observable career outcomes (Petruzziello et al., 2024). Moreover, it supports the conceptualization of psychological capital not as an independent, parallel predictor, but as a dynamic psychological bridge linking human capital to career attainment (Dóci et al., 2023; O'Donovan et al., 2024). The resulting cascade—from motivational energy to relational channels, and finally to realized outcomes—provides critical micro-level evidence for understanding employability generation under complex structural constraints.

Taken together, the established sequential pathway (X → M1 → M2 → Y) not only reconstructs the generative paradigm of employability but also demonstrates that, within China's highly competitive labor market, successful career adaptation does not depend on the accumulation of any single form of capital. Rather, it hinges on the efficiency of capital circulation and cross-domain conversion. This conclusion directly responds to recent theoretical calls emphasizing the processual and malleable nature of employability (Eimer and Bohndick, 2023; Letnar and Širok, 2025).

### 4.2 Measurement invariance and gender-based equifinality

The stepwise establishment of measurement invariance—comprising configural, metric, and scalar invariance—is widely regarded as a prerequisite for meaningful latent mean comparisons (Putnick and Bornstein, 2016), while recent methodological discussions have further emphasized the importance of theory-informed implementation of these tests (Lasker, 2024). Achieving scalar invariance effectively rules out the confounding influence of differential item functioning (DIF), ensuring that observed gender differences in resource distribution reflect substantive group characteristics rather than measurement artifacts.

Empirical results reveal a distinctive pattern of complementary capital allocation within the Chinese student population. Latent mean comparisons indicate that male students exhibit marginal advantages in human capital (X: knowledge and skills), whereas female students demonstrate significantly higher levels of psychological capital (M1: personal qualities). This distribution not only mirrors gender-differentiated socialization processes and role expectations (Letnar and Širok, 2025; O'Donovan et al., 2024) but also reflects gender-specific rational strategies for coping with involutionary labor market pressures. Male students tend to pursue vertical breakthroughs through enhanced technical efficacy, whereas female students display superior emotional regulation, psychological resilience, and socio-emotional resource advantages. Such initial heterogeneity in capital endowments constitutes a structural precondition for subsequent divergence in capital conversion pathways.

More importantly, the study provides strong evidence for gender equifinality in final career development outcomes (Y), thereby substantiating the principle of equivalent endpoints in career development theory (Von Bertalanffy, 1968; Savickas, 2005). Despite markedly different initial capital configurations, male and female students converge toward comparable levels of career development by deploying distinct but functionally equivalent capital mobilization strategies under shared structural constraints.

Specifically, male students' trajectories are characterized by a pronounced human-capital-dependent pathway, wherein accumulated knowledge and skills exert a more direct influence on career development. In contrast, female students exhibit significantly higher capital conversion efficiency: they leverage their psychological capital (M1) as a motivational lever, activating social networks (M2) through more proactive social exploration and interaction, thereby functionally compensating for marginal disadvantages in early-stage human capital accumulation (Petruzziello et al., 2024). This compensatory “psychological–social” mediation pathway demonstrates that employability attainment is not contingent upon dominance in any single dimension but rather upon individuals' ability to flexibly and efficiently reconfigure capital flows in accordance with their unique resource structures.

By challenging unidimensional interpretations of gender advantage, these findings illuminate how individuals achieve equivalent levels of career adaptation through pathway complementarity. This provides a nuanced explanatory framework for understanding how graduates in transitional China attain career success via differentiated yet equally effective forms of agency.

### 4.3 Practical implications

The present findings offer clear evidence-based guidance for higher education institutions (HEIs), underscoring that employability enhancement should move beyond fragmented skill training toward the construction of an integrated “knowledge–psychological–social” empowerment system.

First, university career services must break away from “skill-island” intervention models. The results demonstrate that the ultimate effectiveness of human capital ($X$) is contingent upon its sequential transformation into psychological and social resources. Accordingly, career curricula should extend beyond instrumental guidance such as résumé writing and interview techniques to focus explicitly on cultivating students' capital conversion efficiency. HEIs should introduce social laboratories grounded in proactive behavior theory, systematically guiding students to translate internal career efficacy into concrete social exploration behaviors—such as weak-tie development and informational interviewing—thereby removing bottlenecks between psychological surplus and relational actualization (Granovetter, 1973; Petruzziello et al., 2024; Letnar and Širok, 2025).

Second, HEIs should adopt gender-responsive, resource-based guidance strategies. The demonstrated equifinality of career outcomes reveals differentiated yet effective success pathways across genders. For male students, interventions should prioritize the cultivation of psychological resilience and proactive network building to strengthen the motivational drivers of capital conversion. For female students, institutions should provide higher-level social capital access platforms—such as structured mentoring systems and targeted alumni referral mechanisms—to support upward social capital mobility and enable them to fully leverage their psychological strengths (O'Donovan et al., 2024). Such precision-oriented empowerment strategies can ensure that students from diverse backgrounds achieve optimal resource allocation and sustainable career development.

Beyond these practical applications, a theoretical consideration regarding the sequential vs. parallel nature of the four dimensions deserves clarification. Although they are derived from the same measurement instrument, our empirical results support a serialized path over a simple parallel structure. This suggests that for Chinese undergraduates, future employability is not a static set of traits but a developmental trajectory. Regarding the possibility of reverse paths—such as psychological capital driving knowledge acquisition—while reciprocal effects may exist in long-term professional life, ‘mastery experiences' (human capital) remain the most critical and proximal antecedent of psychological resources for students in the skill-acquisition stage. By adopting this sequential perspective, our model captures the intrinsic ‘resource flow' that parallel models might overlook. Nonetheless, we acknowledge that the cross-sectional nature of this data limits causal inferences, and future longitudinal designs are encouraged to further scrutinize these dynamic interplays. This theoretical consideration leads to several limitations of the current study that warrant further discussion.

### 4.4 Research limitations and future directions

Despite the methodological rigor of the present study, several limitations should be acknowledged. First, the cross-sectional design limits causal inference regarding the temporal ordering among human capital, psychological resources, and career outcomes. Future studies should employ longitudinal or multi-wave designs to examine the dynamic evolution and stability of the proposed sequential conversion structure across different career stages.

Second, all variables were assessed using self-report measures, which may raise concerns about common method variance. Although procedural remedies were applied and the confirmed sequence aligns with established theory, future research should incorporate multi-source data, behavioral indicators, or experimental designs to further strengthen construct validity.

Third, the generalizability of the findings is bounded by the sociocultural and labor-market context of Chinese university students, characterized by intense positional competition and structural constraints. Cross-cultural research is needed to determine whether similar patterns of resource differentiation and cascading conversion emerge in other sociocultural contexts.

Fourth, the present study focused on a specific set of capital dimensions. Future research should integrate objective outcomes, such as starting salaries or job quality, to examine the longer-term implications of the identified pathways.

Finally, while measurement invariance across gender was established, future studies could employ multi-group SEM to formally test whether the path coefficients themselves differ significantly across other social identities or academic disciplines.

## 5 Conclusion

In summary, this study validates a multidimensional measurement model of future employability in a large Chinese undergraduate sample and demonstrates that the instrument functions equivalently across gender. The data reveal a theoretically informative and robust mechanism: knowledge and skills contribute to career development both directly and, critically, indirectly by enhancing personal qualities that facilitate social network formation, which in turn enables career outcomes. The non-significant direct link from personal qualities to career development underscores the vital importance of social embedding for psychological resources to yield career benefits. Methodologically, the ordered pipeline (CFA → measurement invariance → MG-CFA → sequential SEM) offers a rigorous template for future research. Practically, the findings argue compellingly for integrative educational strategies that holistically combine technical training with psychosocial development and network building to foster graduates' sustainable employability in dynamic labor markets.

## Data Availability

The original contributions presented in the study are included in the article/supplementary material, further inquiries can be directed to the corresponding author.
